# Intraperitoneal chemotherapy is now back for ovarian cancer

**DOI:** 10.1007/s10147-025-02700-w

**Published:** 2025-01-29

**Authors:** Keiichi Fujiwara, Shoji Nagao, David Tan, Kosei Hasegawa

**Affiliations:** 1 Department of Gynecologic Oncology, Saitama Medical University International Medical Center, Hidaka, Japan; 2 Department of Obstetrics and Gynecology, Faculty of Medicine, Okayama University, Okayama, Japan; 3 Department of Haematology-Oncology, National University Cancer Institute, Singapore (NCIS), National University Hospital (NUH), Singapore, Singapore

**Keywords:** Ovarian cancer, Intraperitoneal chemotherapy, Carboplatin, Large residual tumor, Safety

## Abstract

The Intraperitoneal Carboplatin for Ovarian Cancer (iPocc) trial demonstrated that intraperitoneal (IP) administration of carboplatin is more effective than intravenous (IV) administration for advanced ovarian cancer, especially in cases with large residual tumors, challenging previous assumptions that IP chemotherapy is only beneficial for small residual tumors. Additionally, the iPocc trial showed that IP chemotherapy has a comparable safety profile to IV chemotherapy, with the exception of port-related toxicities. This review summarizes the principles, development, and significance of IP chemotherapy and discusses its future potential in light of recent studies. Notably, the iPocc trial, conducted under Japan’s new clinical trial regulations, achieving regulatory approval based on investigator-initiated results. The iPocc regimen offers a viable treatment option for patients with advanced ovarian cancer (stages II–IV). However, bevacizumab is recommended for later-line treatments rather than combining it with IP chemotherapy until further trials support such combinations. Future studies are needed to identify biomarkers that predict response to the iPocc regimen. The trial’s success underscores the dedication of patients and families who contributed to this groundbreaking research.

## Introduction

The recently published (Intraperitoneal Carboplatin for Ovarian Cancer, GOTIC-001/JGOG3019/GCIG) trial [1] demonstrated that intraperitoneal (IP) administration of carboplatin, which is the most important cytotoxic agent for ovarian cancer, was more efficacious than intravenous (IV) administration. Notably, the study also showed that IP chemotherapy is more effective than IV chemotherapy for large residual tumors, thus overturning the conventional concept that IP chemotherapy is effective only for small residual tumors that remain after surgery. Furthermore, contrary to the conventional belief that IP chemotherapy is associated with a high rate of adverse events, the safety profile of IP chemotherapy was also shown to be comparable except for port toxicities to that of IV chemotherapy.

To further elucidate the significance of these results from the iPocc trial, this review will recapitulate the principles, history, and importance of IP chemotherapy, and discuss the future of IP chemotherapy in conjunction with data from the most recent studies. We would also like to highlight that the iPocc trial took advantage of Japan’s new clinical trial regulations and led to regulatory approval based on an investigator-initiated clinical trial result.

## Concept of IP chemotherapy

One of the key rationales for IP chemotherapy is that it enables direct exposure of extremely high concentrations of anticancer drugs to IP tumors. In addition, it is expected that the inflow of drugs into the systemic circulation will be suppressed, and as a result, the risks of systemic toxicity will be reduced. Drugs administered intraperitoneally are passively absorbed from the peritoneum into the capillary or lymphatic stream [2, 3], the pharmacokinetics of intraperitoneally administered drugs depend on molecular weight and water solubility (Table 1) [3]. The smaller the molecular weight and the more water-soluble the drug, the more rapidly it is absorbed and the easier it is to enter the systemic circulation. For example, platinum drugs including cisplatin and carboplatin are water-soluble and have a small molecular weight, so they are quickly absorbed through the peritoneum and enter the systemic circulation. As a result, the peritoneal/serum area under the curve (AUC) ratio is lower when these drugs are administered intraperitoneally. Conversely, the taxane agent paclitaxel is non-water-soluble and has a large molecular weight, and remains in the peritoneal cavity for a long time with minimal uptake into the systemic circulation, thus resulting in a higher peritoneal/serum AUC ratio. It has generally been thought that drugs with a high peritoneal/serum AUC ratio, such as paclitaxel, are suitable for intraperitoneal administration.

**Table 1:** Pharmacokinetic characteristics of drugs (from Reference [1] revised)

Drug	Molecular weight	AUC ratio	Water solubility	Penetration depth
Alkylating agents				
Mitomycin C	334.3	13–80	±	2–5 mm
Melphalan	305.2	17–63	−	NA
Platinum compounds				
Cisplatin	300.1	12–22	+	1–5 mm
Carboplatin	371.3	15–20	+	0.5 mm
Oxaliplatin	397.3	16	+	1–2 mm
Topoisomerase inhibitors				
Irinotecan	677.2	15	+	NA
Doxorubicin	580	162–230	±	4–6 cell layers
Anti-microtubule agents				
Paclitaxel	853.9	530–2300	−	> 80 cell layers
Docetaxel	861.9	150–3000	−	1.5 mm
Anti-metabolites				
5-fluorouracil	130.1	117–1400	±	0.5 mm
Gemcitabine	299.6	791–847	±	NA

*AUC* are under the curve, *AUC ratio* intraperito + neal AUC/ plasma AUC

Recently, however, it has become clear that platinum drugs are suitable for IP administration [4]. A pharmacological analysis of platinum concentration in the peritoneal cavity and the serum after the administration of carboplatin showed that the 24 h platinum AUC in the serum was exactly the same regardless of the route administration. On the other hand, 24 h platinum AUC in the peritoneal cavity was about 17 times higher when administered intraperitoneally. This suggests that for drugs that are easily absorbed into the systemic circulation, such as platinum drugs, IP administration may provide both higher intraperitoneal drug concentrations while facilitating drug concentrations in the systemic circulation that are equivalent to IV administration.

The direct penetration of anticancer drugs into tumors within the peritoneal cavity is thought to be several millimeters at most. Therefore, patients who achieved complete surgical resection or had a small residual tumor were targeted for IP chemotherapy. Water-soluble drugs, however, with a small molecular weight can be expected to reach the tumor both directly from the peritoneal cavity and via the tumor’s feeding blood vessels through the systemic circulation, and may, thus, be effective regardless of the size of the tumor.

Another consideration for drugs administered intraperitoneally is that they have less peritoneal irritation. Paclitaxel is a relatively strong peritoneal irritant. In the GOG 172 trial, which included intraperitoneal administration of paclitaxel, many patients developed catheter related complication, and only about 42% of patients completed planned 6 cycles of chemotherapy [5, 6].

## History of IP chemotherapy

Intraperitoneal chemotherapy, which was proposed approximately 30 years ago, has been extensively studied in both preclinical and clinical studies, and its usefulness has been demonstrated [5]. In 2005, the US National Cancer Institute (NCI) and Gynecologic Oncology Group conducted a meta-analysis of seven randomized controlled trials that had been completed until then, and found that intraperitoneal chemotherapy reduced the risk of death by 21.6% [7]. This important information was released as a clinical announcement from NCI in January 2006. This announcement indicated that intraperitoneal administration of cisplatin or cisplatin and paclitaxel should be considered for stage III epithelial ovarian cancer without residual tumor after radical treatment.

Despite this recommendation, IP chemotherapy has not been widely accepted in clinical practice [8]. There are several reasons for this avoidance of IP chemotherapy. First, there is uncertainty regarding IP chemotherapy efficacy related to trial design issues, such as imbalance in drug dosage and lack of comparison with standard chemotherapy. Second, there are concerns about cisplatin toxicity and peritoneal irritation caused by IP administration of paclitaxel. Third, there are concerns about the IP port which includes fear of port related complications and their subsequent management.

Several study groups initiated clinical trials, with the intention of addressing these issues with improved trial designs [9–11]. These include the GOG252 study conducted by GOG in the United States, the OV21/PETROC study, an international joint study conducted mainly in Canada and Europe, and the iPocc study, an international joint study conducted mainly in Japan.

**Fig. 1:**
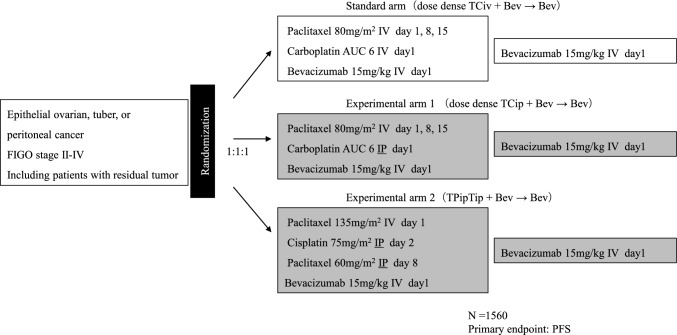
GOG252 study design. *IV* intravenous administration, *IP* intraperitoneal administration

1. GOG252 study (Fig. 1) [9].

The GOG252 study targeted women with primary treatment for epithelial ovarian cancer in FIGO stage II–III, including suboptimal cases with residual tumors of 1 cm or more in maximum diameter after primary cytoreductive surgery (PDS), and 1560 patients were enrolled. Bevacizumab was administered as concomitant and maintenance therapy in all arms. The median PFS for ddTCiv + Bev → Bev therapy, ddTCip + Bev → Bev therapy, and TPipTip + Bev → Bev therapy was 24.9 months, 27.4 months, and 26.2 months, respectively. The hazard ratios of ddTCip + Bev → Bev therapy and TPipTip + Bev → Bev therapy for ddTCiv + Bev → Bev therapy were 0.925 (95% CI 0.802–1.07) and 0.977 (95% CI 0.847–1.13), respectively, and there was no significant improvement in PFS. Similarly, no improvement in overall survival (OS) was observed.

**Fig. 2:**
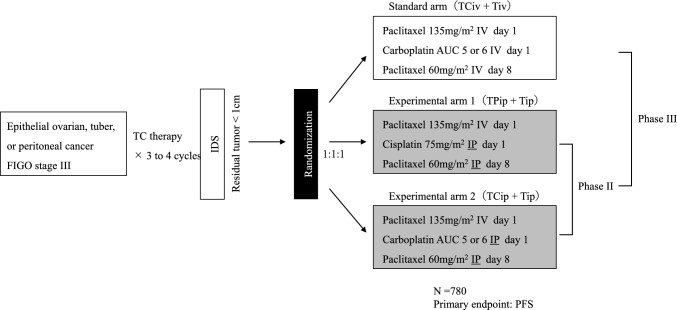
OC21/PETROC study design. TC therapy: paclitaxel plus carboplatin combination therapy, *IDS* interval debulking surgery, *IV* intravenous administration, *IP* intraperitoneal administration. This study uses a pick-up the winner strategy. The winner arm in the phase II part will be compared to standard arm in the phase III part

2. OV21/PETROC study (Fig. 2) [10].

The OC21/PETROC study targets women with FIGO stage III epithelial ovarian cancer who have received interval debulking surgery (IDS) after neoadjuvant chemotherapy (NAC) (3 to 4 cycles of TC therapy) to reduce residual tumor to less than 1 cm. This study was planned as a phase II/III study. First, TCip + Tip therapy and TPip + Tip therapy were compared in the phase II part; subsequently, the winner and the standard treatment are compared in the phase III part. TCip + Tip therapy, which was less toxic, was chosen as the winner arm in the phase II portion of the study, but could not complete the phase III study due to lack of funding. There was a trend toward less progression at 9 months with TCip + Tip therapy, 38.6 vs. 24.5% (*P* = 0.065). In addition, the hazard ratios for PFS and OS were 0.82 (95% CI 0.57–1.17, *P* = 0.27) and 0.80 (95% CI 0.47–1.35, *P* = 0.40), respectively, and no significant difference was observed due to insufficient power.

**Fig. 3:**
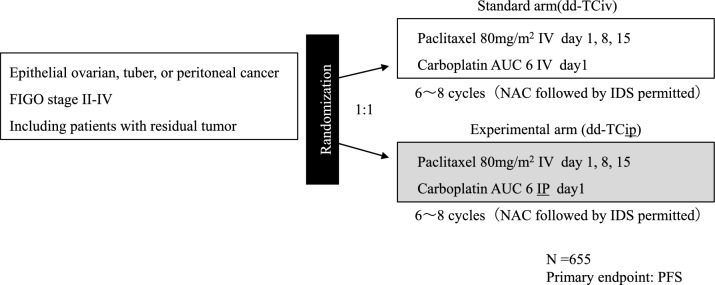
iPocc study design. *IV* intravenous administration, *IP* intraperitoneal administration, *NAC* neoadjuvant chemotherapy, *IDS* interval debulking surgery

3. iPocc trial (Fig. 3) [1, 11].

The iPocc trial purely compared ddTCiv therapy and ddTCip therapy among FIGO stage II to IV epithelial ovarian cancer, and all conditions other than the administration route of carboplatin are the same. Similar to the GOG252 study, it also included suboptimal patients after PDS and allowed IDS after 3 to 5 cycles of NAC. This study was based on two hypotheses: (1) carboplatin IP improves survival of patients with advanced epithelial ovarian cancer when combined with weekly intravenous paclitaxel 80 mg/m^2^ compared to intravenous carboplatin, and (2) IP therapy improves the prognosis of suboptimal patients after PDS. Median PFS was 20.7 months with ddTCiv therapy vs. 23.5 months with ddTCip therapy, hazard ratio 0.83 (95% CI 0.69–0.99, *P* = 0.041), and IP carboplatin significantly improved PFS, for more than 5-10 years. In subgroup analysis, ddTCip therapy tended to have a favorable prognosis regardless of residual tumor size, FIGO stage, age, and PS. The median OS was 67.0 months for ddTCiv therapy versus 64.9 months for ddTCip therapy, with a hazard ratio of 0.95 (95% CI 0.77–1.17, *P* = 0.41), and no improvement in prognosis was observed with IP carboplatin. All grade abdominal pain (34.7 vs. 51.7%), catheter infection (0.7 vs. 10.1%), and vaginal stump leakage (0.3 vs. 5.7%) were more common in ddTCip therapy, but there was no difference in the incidence of grade 3 or higher toxicity: 96.0 vs. 93.2%.

## What is the optimal treatment strategy of iPocc regimen (without TRiPocc results)?

Based on the subset analysis results, iPocc regimen should be considered for all patient population regardless of the size of residual tumor after initial surgery. Even in patients who are considered to receive neoadjuvant chemotherapy, this regimen can be used.

Subgroup analysis from the GOG252 trial indicated that use of bevacizumab may eliminate the benefit of IP carboplatin therapy, although the underlying mechanism for this observation is unclear. Likewise, it remains unclear whether the benefit of the iPocc regimen is retained in the context of maintenance therapy with PARP inhibitors. Further well-designed randomized phase 3 trials will be required to address these controversies.

Nevertheless, the most important observation from the iPocc trial result is the fact that the PFS curves has never crossed for 5–10 years. This implies that there are approximately 10% more patients who survived without recurrence in IP arm compared with patients with IV arm and highlights the potential benefit of IP carboplatin therapy especially in regions where the access to and affordability of drugs such as bevacizumab or PARP inhibitors remain limited.

## Brief summary of TRiPocc study

A key question following the results of for the iPocc trial is the issue of how to best select ovarian cancer patients for IP carboplatin therapy. The better the molecular determinants were understood that may help to stratify patients for IP therapy, patient samples from the iPocc study were collected and subjected to mRNA profiling (Affymetrix HTA 2.0) and ACT Genomics ACTOnco^®^ + next generation sequencing. Cox regression analyses were performed to identify genes significantly correlated with PFS following IP treatment (*P* < 0.05) [12]. A 72-gene signature was significantly correlated with PFS in IP but not in IV patients. A low IP resistance score (IPRS) based on the signature was associated with better outcomes following IP chemotherapy. A high IPRS was significantly correlated with poorer PFS after IP therapy (median: not reached/NR vs ~ 18 months, *P* < 0.0001), with no correlation seen after IV therapy (median: 25 vs 24 months, *P* = 0.9). Low IPRS IP patients had improved PFS compared with low IPRS IV patients (median: NR vs 25 months, *P* = 0.0261). Paradoxically, when IPRS is high, IP patients have worse PFS than IV patients (median: ~ 18 vs 24 months, *P* = 0.004). Tumors with reduced VEGFA/VEGFR2 and PI3K signaling were associated with a low IPRS and were associated with improved outcomes for IP therapy. Notably, there was no difference in IPRS between BRCA1/2 wildtype and mutant tumors (*P* = 0.55), and HRD positive tumors had a significantly higher IPRS than non-HRD tumors (*P* = 0.03).

In murine xenograft models, IP chemotherapy has been shown to induce an antitumor immune response and enhance survival [13]. To investigate whether the immunological milieu influences clinical outcomes in patients undergoing IP chemotherapy, immune profiling of tumor specimens obtained at the time of primary surgery prior to chemotherapy from participants enrolled in the iPocc P3 trial was performed [14]. Cell types within the tumor microenvironment were quantified using the microenvironment cell populations (MCP) counter. Subsequently, single-sample Gene Set Enrichment Analysis was conducted to assess the tumor immune microenvironment. A cohort of 116 patients was included in this analysis, comprising 59 and 57 patients in the IP and IV treatment arms, respectively. Patients receiving IP therapy exhibited prolonged OS compared to those receiving IV therapy in cohorts demonstrating high infiltration of T cells, natural killer (NK) cells, or cytotoxic lymphocytes as determined by the MCP counter (median OS: not reached (NR) vs 75.0 months, *P* = 0.041; NR vs 75.0 months, *P* = 0.042; NR vs 65.2 months, *P* = 0.031), whereas no significant difference was observed in cohorts with low infiltration. Similarly, IP therapy conferred improved OS in patients exhibiting high expression of immune-related genes such as *CD8A*, *FOXP3*, or *PDCD1* (median OS: NR vs 65.2 months, *P* = 0.035; NR vs 65.2 months, *P* = 0.030; NR vs 53.1 months, *P* = 0.016), with no discernible benefit observed in patients displaying low gene expression levels. Furthermore, patients were stratified into “Immune Hot” and “Immune Cold” groups based on hierarchical clustering analysis utilizing parameters representing “Innate immunity,” “T cells,” “Interferon-gamma response,” and “Inhibitory molecules.” Notably, individuals receiving IP therapy demonstrated improved PFS and OS compared to those receiving IV therapy within the “Immune Hot” subgroup (median PFS: 35.5 vs 23.4 months, *P* = 0.024; median OS: NR vs 75.0 months, *P* = 0.040), whereas no significant difference was observed in the “Immune Cold” cohort. These findings suggest that IP carboplatin therapy may enhance survival outcomes in epithelial ovarian cancer patients exhibiting a pre-treatment tumor microenvironment characterized by the “Immune Hot” phenotype.

Both the IPRS score and immune signatures will need to be validated in larger prospective or retrospective cohorts of patients who have been subjected to IP carboplatin therapy.

## Future directions

As mentioned earlier, the role of IP carboplatin therapy in the era of PARP inhibitor will need to be better defined. Furthermore, given the recent positive data on HIPEC, it is important to explore the relationship of IP carboplatin therapy with HIPEC with cisplatin [15]. The use of HIPEC remains controversial [16], and it remains to be determined whether HIPEC with cisplatin is better than IP carboplatin alone, or whether carboplatin can be incorporated into HIPEC. Finally it will be crucial to validate the biomarkers that can predict the efficacy of IP chemotherapy for patients with advanced OC.

## Ovarian cancer treatment guideline coverage

As of July 2024, NCCN guideline included iPocc regimen, and we are waiting for the discussion part. Therefore, technically iPocc regimen will be provided to patients in countries or regions where NCCN guideline recommendations can be clinically implemented.

For Treatment Guideline for Ovarian Cancer by Japanese Society of Gynecologic Oncology, the part of IP chemotherapy will be amended in 2025. However, in Japan, we have to wait the governmental approval for health insurance coverage so that reimbursement is obtained.

## Regulatory imprecations of iPocc trial results in Japan, for IP carboplatin as a coverage by the Japanese Health Insurance System

In Japan, approval is required under the National Health Insurance System with respect to medical insurance reimbursement. In the past, regulatory approval for drug therapies was only granted by pharmaceutical companies, but in 2004, an investigator-initiated indication-directed trial (IIIDT) system was established to legally allow researchers to conduct indication-directed trials (so-called Chiken) for the purpose of applying for approval. However, the regulatory requirements for IIIDT are extremely high, and further deregulation has been desired. To accomplish this flexibility, the government introduced the Advanced Medical Care System and enacted the Clinical Research Act to enable pharmaceutical approval based on the results of high-quality, investigator-initiated clinical trials (2023).

The iPocc trial was conducted in 2009 with the approval of the Ministry of Health, Labour and Welfare as “an Evaluation System of Investigational Medical Care” (later called “Advanced Medical Care”), and has been conducted as a Specified Clinical Trial after the enactment of the Clinical Research Act. We hope that this treatment will be available to ovarian cancer patients in Japan as soon as possible.

By the way, if the iPocc Regimen is approved, it will be the first pharmaceutical approval in Japan based on the results of an investigator-initiated international phase III study.

Furthermore, from the beginning of the trial’s operation, budgetary measures have been taken so that 100,000 yen per case will be allocated to domestic and overseas facilities to be used for supporting researchers. Although this is not a sufficient amount of funding, it is highly possible that this has made international joint trials possible.

## Summary

In summary, iPocc regimen is a reasonable treatment choice of all advanced ovarian cancer patients regardless of residual disease size with stages IIIV. Bevacizumab should not be combined with iPocc regimen and should be preserved for later line in case of recurrence, until the new trials demonstrate the benefit of bevacizumab combining with IP chemotherapy. Incorporation of PARP inhibitors with iPocc regimen should be prospectively studied. Furthermore, studies to validate biomarkers that predict the benefit of iPocc regimen should be explored. Most importantly, the results of the study are also a testament to the courage of our patients and their families to whom we would like to express our deepest gratitude for their cooperation in the iPocc trial.
